# Microenvironmental Influence on Pre-Clinical Activity of Polo-Like Kinase Inhibition in Multiple Myeloma: Implications for Clinical Translation

**DOI:** 10.1371/journal.pone.0020226

**Published:** 2011-07-07

**Authors:** Douglas W. McMillin, Jake Delmore, Joseph Negri, Melissa Ooi, Steffen Klippel, Chandrasekhar V. Miduturu, Nathanael S. Gray, Paul G. Richardson, Kenneth C. Anderson, Andrew L. Kung, Constantine S. Mitsiades

**Affiliations:** 1 Jerome Lipper Multiple Myeloma Center, Department of Medical Oncology, Dana-Farber Cancer Institute, Boston, Massachusetts, United States of America; 2 Department of Medicine, Harvard Medical School, Boston, Massachusetts, United States of America; 3 Department of Biological Chemistry and Molecular Pharmacology, Dana-Farber Cancer Institute, Department of Cancer Biology and Harvard Medical School, Boston, Massachusetts, United States of America; 4 Department of Pediatric Oncology, Dana-Farber Cancer Institute and Children's Hospital Boston, Boston, Massachusetts, United States of America; Clinica Universidad de Navarra, Spain

## Abstract

Polo-like kinases (PLKs) play an important role in cell cycle progression, checkpoint control and mitosis. The high mitotic index and chromosomal instability of advanced cancers suggest that PLK inhibitors may be an attractive therapeutic option for presently incurable advanced neoplasias with systemic involvement, such as multiple myeloma (MM). We studied the PLK 1, 2, 3 inhibitor BI 2536 and observed potent (IC50<40 nM) and rapid (commitment to cell death <24 hrs) *in vitro* activity against MM cells in isolation, as well as *in vivo* activity against a traditional subcutaneous xenograft mouse model. Tumor cells in MM patients, however, don't exist in isolation, but reside in and interact with the bone microenvironment. Therefore conventional in vitro and in vivo preclinical assays don't take into account how interactions between MM cells and the bone microenvironment can potentially confer drug resistance. To probe this question, we performed tumor cell compartment-specific bioluminescence imaging assays to compare the preclinical anti-MM activity of BI 2536 *in vitro* in the presence vs. absence of stromal cells or osteoclasts. We observed that the presence of these bone marrow non-malignant cells led to decreased anti-MM activity of BI 2536. We further validated these results in an orthotopic *in vivo* mouse model of diffuse MM bone lesions where tumor cells interact with non-malignant cells of the bone microenvironment. We again observed that BI 2536 had decreased activity in this *in vivo* model of tumor-bone microenvironment interactions highlighting that, despite BI 2536's promising activity in conventional assays, its lack of activity in microenvironmental models raises concerns for its clinical development for MM. More broadly, preclinical drug testing in the absence of relevant tumor microenvironment interactions may overestimate potential clinical activity, thus explaining at least in part the gap between preclinical vs. clinical efficacy in MM and other cancers.

## Introduction

One of the problems in oncology drug development today is the discordance of highly promising *in vitro* and *in vivo* preclinical results with the lack of efficacy observed in patients. Less than 8% of agents that enter phase I clinical trials in cancer eventually become FDA approved for the treatment of any tumor type [1]. Data from our group and others indicate that interaction of malignant cells with their local microenvironment can confer drug resistance, which may account for this gap between the preclinical drug activity and clinical efficacy [2], [3].

Polo-like kinases (PLKs) are particularly interesting targets in cancer because of their role in cell cycle progression, checkpoint control and mitosis [4], [5]. Tumors with PLK overexpression are associated more frequently with chromosomal instability, DNA aneuploidy and centrosome amplification [6]. In addition, cancer cells are more sensitive to PLK inhibition than normal cells [7]; and PLK expression has been shown to be higher in cancer cells with a high mitotic index [8]. In advanced multiple myeloma (MM), malignant cells have a high mitotic index [9] and chromosomal instability [10], suggesting that PLK inhibitors may be an attractive therapeutic option for this presently incurable disease.

Here we evaluate the activity of the PLK 1, 2, 3 inhibitor BI 2536 in preclinical models of MM and investigate the role of the microenvironment in modulating its anti-MM activity. We observed potent anti-MM activity in traditional drug development experiments, but decreased activity of BI 2536 in bone microenvironmental models. Our results highlight that BI 2536 represents a compound with promising characteristics, but its lack of activity in microenvironmental models of MM raises concerns for its clinical development for this disease. These concerns are compatible with the limited clinical activity that this agent has shown so far in clinical trials in solid tumors, even though clinically achievable concentrations exceed the levels needed for *in vitro* anti-tumor activity based on conventional models. These models can overestimate the drug activity because they do not incorporate tumor-microenvironment interactions. More broadly, our study provides a concrete example of the importance of preclinical testing of investigational therapeutics in models that simulate how the non-malignant accessory cells of the tumor microenvironment may confer drug resistance.

## Results and Discussion

### Anti-MM activity of PLK inhibitor *in vitro* and *in vivo* in the absence of bone microenvironmental interactions

**Figure 1:**
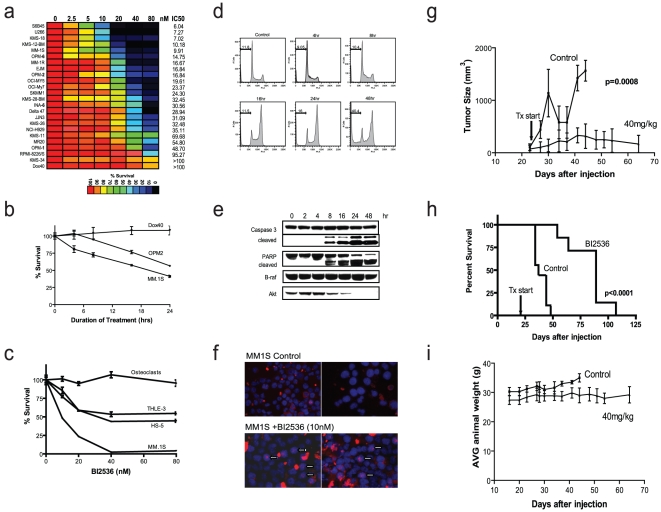
Activity of the small molecule PLK1, 2, and 3 inhibitor BI 2536 in preclinical models of MM in the absence of the bone microenvironment. (A) BI 2536 (0–80 nM; 72 hrs) was tested against a panel of MM cell lines by MTT assay, the majority having IC50 values <40 nM. (B) Dox40, OPM2, and MM.1S were evaluated by cell death commitment assay (0–24 hrs; 50 nM drug exposure, followed by 72 hrs of culture in drug-free media). MM.1S and OPM2 (sensitive cell lines from panel A) required <24 hr of exposure to BI 2536 to commit cells to death. In contrast, up to 24 hr of drug exposure had no effect on Dox40 viability. (C) Non-malignant cells, including immortalized hepatocytes (THLE-3) and stromal cells (HS-5), as well as osteoclasts (OC), were evaluated for sensitivity to BI 2536 (72 hrs) by MTT assay. All non-malignant cells tested remain less sensitive to the drug than the majority of MM cell lines. (D) Cell cycle analysis of KMS18 cells exposed to BI 2536 (10 nM; 4–48 hrs) shows pronounced increase in the G2/M events, followed by an increase in subG_0_/G_1_ events at later time points. (E) Western blot analysis revealed cleavage of caspase 3 and PARP as early as 8 hrs of drug exposure (20 nM), indicating apoptotic cell death in KMS18 cells. Decreases in Akt, but not B-raf, were observed in response to treatment. (F) MM.1S cells were exposed to 10 nM BI 2536 for 24 hrs, stained for α-tubulin (Red) and Hoechst (Blue) and compared to non-treated controls. Treatment with BI 2536 caused an increase in monopolar aster formation as a result of the effects on cell cycle progression and division. (G–I) Animal studies performed in a subcutaneous model of MM (n = 9, control n = 8, treated) showed significant suppression of tumor burden (p = 0.0005; panel G), prolongation of survival (p<0.0001; Kaplan-Meier & log rank test; panel H) and no significant decrease in average body weight (panel I), consistent with lack of major toxicity. The transient decrease in average tumor burden of control mice is due to the early death of some mice with high tumor burden (G).

Because of the activity of BI 2536 in other cancer models and the role of PLKs in cell cycle regulation [4], [5], we evaluated a panel of MM cell lines for sensitivity to BI 2536 (Fig. 1a). We observed potent activity with IC_50_ values <40 nM for the majority of cell lines, including lines resistant to established anti-MM agents (e.g. dexamethasone-resistant MM.1R). The BI 2536 concentrations required for *in vitro* anti-MM activity are within its clinically achievable levels [11]. These *in vitro* responses were rapid, requiring <24 hrs of drug exposure to commit cells to death (Fig. 1b). Non-malignant cells, such as HS-5 stromal cells, THLE-3 hepatocytes and osteoclasts (OCs) had IC_50_ values >40 nM (Fig. 1c). The potency and rapid kinetics of BI 2536 activity are also highlighted by the cell cycle analysis (G2/M arrest, followed by increase sub-G0 events; Fig. 1d), rapid cleavage of caspase 3 and PARP within 8 hrs of treatment (Fig. 1e) and formation of monopolar asters (Fig. 1f). In a subcutaneous animal model, BI 2536 significantly suppressed tumor burden (Fig. 1g) and prolonged survival (Fig. 1h), without changes in body weight (Fig. 1i). These preclinical *in vitro* and *in vivo* data are similar to those that provided in the past the framework for further clinical development of BI 2536 in other neoplasias. These experiments in other models, however, did not take into account the ability of the bone microenvironment to modulate drug activity.

### Effects of the bone microenvironment on PLK inhibitor activity *in vitro* and *in vivo*

Emerging literature indicates that the activity of different anti-cancer agents can be modulated by the non-malignant accessory cells of the microenvironment in which tumor cells are located [2]. We therefore recently developed an *in vitro* model, tumor cell compartment-specific bioluminescence imaging (CS-BLI), which allows high-throughput scalable evaluation of investigational agents in co-cultures simulating the tumor-stromal interactions [2]. This allows us to identify compounds with increased, as well as others with deceased, antitumor activity in the presence of stromal cells [2]. Importantly, we used of this in vitro co-culture platform in tandem with our orthotopic animal models and validated *in vivo* these observations in conditions which reflect the tumor microenvironment and simulate the patient condition [2].

**Figure 2:**
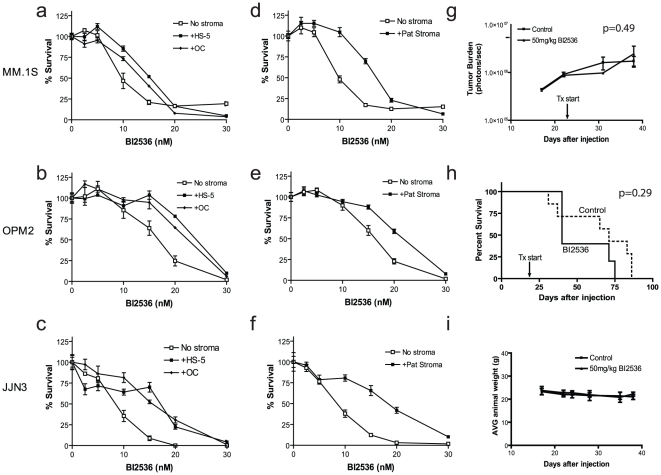
Anti-MM activity of BI 2536 is attenuated in preclinical models incorporating MM cell interaction with the bone microenvironment. (A–C) MM.1S, OPM2 and JJN3 cells were tested in the presence and absence of HS-5 stromal cells and differentiated OC of the bone. Co-culture with stromal cells and OC decreases the sensitivity of MM cells to BI 2536. (D–F) Stromal cells isolated from MM patients also decrease the sensitivity of MM.1S, OPM2, and JJN3 cells to BI 2536. (G–I) Animal studies performed in the MM model of diffuse lesions in the bone (n = 8, control n = 7, treated) did not show significant changes in tumor burden (p = NS; panel G), prolongation of survival (panel H) or decrease in average body weight (p = NS; panel I).

Based on this experience, we assessed the effect of the bone microenvironment on the anti-MM activity of BI 2536. We first tested the compound *in vitro* against MM cell lines cultured alone or in the presence HS-5 stromal cells, differentiated OCs or primary bone marrow stromal cells from MM patients. Drug activity was selectively assessed using the CS-BLI assay. BI 2536 was less active against MM.1S (Fig. 2a), OPM2 (Fig. 2b) and JJN3 (Fig. 2c) MM cells in the presence of HS-5 stromal cells or differentiated OCs compared to the absence of accessory cells. In addition, BI 2536 was less active against MM.1S (Fig. 2d), OPM2 (Fig. 2e) and JJN3 (Fig. 2f) cells in the presence of patient stroma. The shift in IC_50_ in the presence of accessory cells indicates a smaller difference in IC_50_ compared to stromal cells and hepatocytes, which suggests there may be a reduced therapeutic window for this compound in the presence of elements of the bone milieu.

In addition to our *in vitro* microenvironment assessment of BI 2536, we tested the compound in our *in vivo* orthotopic model of diffuse myeloma lesions [12]. In this model, tumor cells home to the bone in multiple sites, consistent with the clinical presentation of myeloma in patients. We observed no significant activity of BI 2536 when administered at 50 mg/kg 2x weekly (Fig. 2g) and no significant prolongation of survival in treated mice vs. vehicle treated controls (Fig. 2h). Although this dose was higher than in the s.c. model, we still did not observe significant toxicity, as evidenced by the lack of weight loss in the BI 2536 treated mice compared to vehicle-treated controls (Fig. 2i). The MM.1S cell line model utilized in our *in vivo* studies was one of the most drug-responsive cell lines *in vitro*, however, its *in vivo* response in the s.c. model, but not the diffuse bone model, illustrates that tumor cells highly sensitive to a given drug in the absence of the bone microenvironment may become more drug-resistant in its presence.

One of the biggest problems in oncology remains the low success rate of translating preclinical results to effective clinical drugs. Among the various reasons for this discrepancy, our data suggest that the tumor microenvironment plays a role in decreasing anti-cancer drug efficacy. PLK inhibitors such as BI 2536, although active against tumors studied outside the context of their bone microenvironment, are less active in models that take this interaction into account. The application of both *in vitro* and *in vivo* models that simulate how tumors interact with the microenvironment in patients would improve the prioritization of PLKs inhibitors, and other compounds, for further development. Utilizing orthotopic *in vivo* models, which include all the elements of the local tumor microenvironment, in conjunction with co-culture *in vitro* screening offers a better simulation of clinical condition than conventional models. Relying exclusively on *in vitro* co-culture assays, especially if supra-pharmacological doses and non-relevant durations of exposure are used, may over-estimate the ability of a given drug to overcome microenvironment-dependent drug resistance. This may explain why prior studies using BI5236 at 2.5 µM (a dose higher than the Cmax in patients) did not detect *in vitro* stroma-induced resistance of tumor cells[13].

Interestingly, multiple clinical trials of BI 2536 in solid tumors [11], [14], [15], [16] have shown limited objective clinical responses in clinical trials, despite achievable levels of BI 2536 far exceeding those showing in vitro activity in solid tumor models. This raises the possibility that, similarly to our observations in MM models, solid tumor cells may also exhibit a microenvironment-dependent resistance to BI 2536. Furthermore, other clinical trials of agents in the same class have shown limited clinical activity as well [17], suggesting that the lack of clinical activity so far for this drug class could be attributable in part to microenvironmental factors, although further studies are warranted.

**Figure 3:**
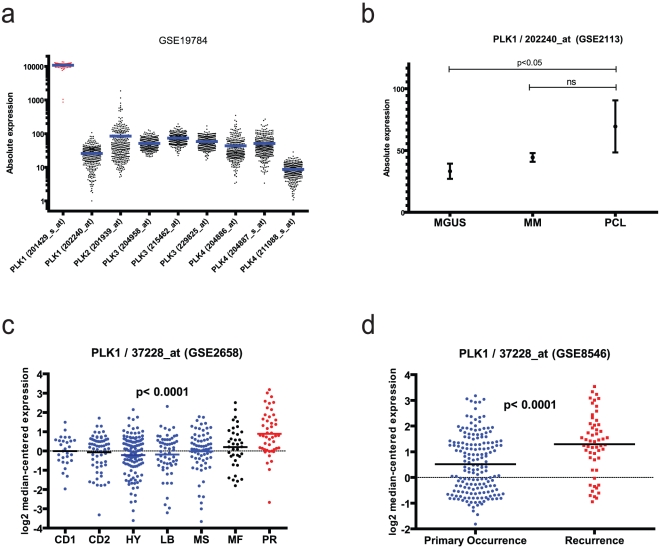
Clinical relevance of PLK in patients from the HOVON myeloma dataset. (A) Myeloma patient samples from the Dutch HOVON trial (N = 320, GSE19784) were evaluated for the expression of PLK transcripts. PLK1 (201429_at) is expressed at higher levels in patients compared to the other PLK isoforms. (B) We observe a significantly higher expression level of PLK1 transcript (202240_at) in PCL patients compared to MGUS (P<0.05) and a trend for higher expression in MM vs. MGUS patients in the GSE2113 dataset; (C) Expression of PLK1 transcript is higher among newly diagnosed MM patients which are classified to have a proliferative (PR) transcriptional signature in their CD138+ tumor cell compared to other gene expression-based molecular subtypes of MM, specifically the CD1, CD2, HY, LB, and MS subtypes (GSE2658 dataset). (D) The expression of PLK1 transcript is higher in MM patients when disease recurrence is observed compared to pre-treatment samples (GSE8546 dataset).

Although we observed microenvironment resistance to BI 2536 in preclinical models, there may be subpopulations of MM patients who may respond to this drug despite these microenvironment interactions. To evaluate this we examined the gene expression data of myeloma patients from the Dutch HOVON trial (N = 320) for the expression of PLK transcripts. We observed that PLK1 (201429_at) is expressed at higher levels in patients compared to the other PLK isoforms (Fig. 3a). Comparing PLK1 transcript (202240_at) in various stages of disease, we observe a significantly higher expression level in plasma cell leukemia (PCL) patients compared to individuals with the premalignant monoclonal gammopathy of undetermined significance (MGUS) (P<0.05) and a trend compared to MM patients, although not statistically significant (Fig. 3b). Interestingly, we observed significantly higher PLK1 transcript (37228_at) expression in the proliferative (PR) subtype of MM compared to CD1 (cases with *CCND1* upregulation because of t(11;14) translocation), CD2 (cases with *CCND3* upregulation because of t(6;14) translocation), HY (hyperdiploid MM), LB (“Low Bone” disease group, characterized by low number of magnetic resonance imaging (MRI)–defined focal bone lesions and low expression of DKK1), and MS (cases with MMSET overexpression due to t(4;1;4) translocation) subtypes (1-way ANOVA; p<0.0001; Dunn's multiple comparison post-hoc tests for each of the comparison between subtypes, p<0.05), but not compared to the MF subtype (cases with overexpression of *MAF/MAFB*) of MM patients (p>0.05; Fig. 3c). The PR subtype of MM is the one that most closely resembles the molecular profiles of MM cell lines [18], such as those evaluated in our study. This observation suggests that PLK inhibition may still be an interesting putative therapeutic approach for MM patients harboring a proliferative transcriptional signature in their tumor cells. However, our observations that BI 2536 is subject to microenvironment-dependent drug resistance suggest that caution is warranted for further clinical development in MM, even for patients of the PR subtype.

Preclinical models currently applied in most cancers don't take into account tumor microenvironmental interactions, and therefore may overestimate the clinical activity of investigational agents. Incorporating the microenvironmental component into preclinical studies may reconcile the discordance between preclinical and clinical efficacy and thereby improve the bench to bedside translation of effective therapies. In conclusion, BI 2536 is a less attractive agent for development in MM based on our microenvironment data, unless further studies determine clinically applicable methods to overcome this microenvironment-dependant resistance.

## Materials and Methods

### Compound

BI 2536 (CAS 755038-02-9, C_28_H_39_N_7_O_3_, MW 521.66) was synthesized by published methods [19], characterized by ^1^H and ^13^C nuclear magnetic resonance, solubilized in DMSO (in vitro) or 0.1N HCl/0.9% NaCl (*in vivo*) and used as indicated in figure legends.

### Cell viability assays

Viability of MM cell lines, THLE-3 hepatocytes, and HS-5 stromal cells was measured by 3-(4,5-dimethylthiazol-2-yl)-2,5-diphenyl tetrasodium bromide (MTT; Chemicon International) colorimetric assay, as previously published [20], and viability of osteoclasts (OC) was measured by CellTiterGlo assay (CTG; Promega) for 72 hrs. *In vitro* osteoclast differentiation from normal donor PBMCs was performed as previously described [21]. For cell death commitment assays, MM cells were exposed to BI 2536 (20 nM) for up to 24 hrs, washed and incubated in drug-free medium for 3 days and quantified by MTT assay. For co-culture experiments, tumor compartment specific-bioluminescence imaging (CS-BLI) was used to selectively detect tumor cell viability[2].

### Cell cycle analysis

KMS18 cells treated with BI 2536 (20 nM; 0–48 hrs) were stained using propidium iodide/RNase A (Sigma) following 70% EtOH fixation, passed through a flow cytometer (Beckman Coulter) and analyzed using FlowJo software (Treestar).

### Immunoblotting and immunoflourescence studies

KMS18 cells were treated with BI 2536 (20 nM) for 0–48 hrs and immunoblotted, as previously described [22]. For immunoflourescence studies, cells were fixed in 2% Paraformaldehyde; resuspended in 5% Goat Serum (GS) in PHEM buffer and cytospun onto coverslips; MeOH permeablized, blocked with 10% GS/PHEM and incubated with primary/secondary antibodies and Hoechst counter-stain; mounted with Vinol mounting media and imaged using a Nikon E800 Eclipse Microscope (Nikon, Japan) and Cool Snap HQ2 Camera (Photometrics).

### *In vivo* anti-tumor activity of BI 2536

Sublethally irradiated (150 rads) CB17-SCID mice were injected with MM1.S-GFP/luc cells subcutaneously (2.5×10^6^) or i.v. (10^6^). Following engraftment, mice received i.v. BI 2536 (40 mg/kg, s.c. model or 50 mg/kg i.v. model 2x weekly). Mice were monitored regularly for changes in tumor burden (calipers for subcutaneous model and Xenogen IVIS system for i.v. model), changes in body weight and sacrificed in accordance with institutional guidelines. Mice were housed in the Animal Research Facility of the Dana-Farber Cancer Institute and experiments were performed in accordance with protocol approved by the Dana-Farber Cancer Institute Animal Care and Use Committee (ACUC) (protocol #04-111) and in accordance with relevant national and international guidelines, including steps taken to ameliorate any suffering of animals.

#### Expression of PLK transcripts in MM patient cells

Gene expression data from publicly available Gene Expression Omnibus (GEO) datasets (accession numbers GSE19784, GSE2113, GSE2658 and GSE8546) were downloaded and analyzed through Oncomine 4.4 (for datasets GSE2658 and GSE8546) or directly from GEO (datasets GSE19784 and GSE2113). These datasets included profiles of CD138+ myeloma cells from patients enrolled in the Dutch HOVON trial (N = 320, GSE19784) [23]; CD138+ plasma cells from patients with monoclonal gammopathy of undetermined significance (MGUS), myeloma or plasma cell leukemia (7, 39 and 6 cases respectively, N = 52 total, GSE2113) [24]; CD138+ myeloma cells from 414 newly diagnosed MM patients (GSE2658) [25]; and CD138+ myeloma cells from 174 cases prior to initiation of therapy vs. 55 cases after disease recurrence (N = 229 total, GSE8546) [26]. Using one-way ANOVA analysis, we evaluated the absolute expression of various probes for PLK1, 2, 3 and 4 (GSE19784); the differences in expression of PLK1 transcript in MGUS, MM and PCL (GSE2113); as well as the differences in log_2_-transformed median-centered transcript levels, in the GSE19784 dataset [23] for PLK1 in various molecularly defined subtypes of MM [25]. The difference in log_2_-transformed median-centered transcript levels of PLK1 in primary vs recurrent disease (GSE8546) was evaluated by unpaired t-test. Statistical analyses were performed with Prism 5 software (Graphpad).
